# Defoliation Significantly Suppressed Plant Growth Under Low Light Conditions in Two Leguminosae Species

**DOI:** 10.3389/fpls.2021.777328

**Published:** 2022-01-07

**Authors:** Ning Wang, Tianyu Ji, Xiao Liu, Qiang Li, Kulihong Sairebieli, Pan Wu, Huijia Song, Hui Wang, Ning Du, Peiming Zheng, Renqing Wang

**Affiliations:** ^1^Institute of Ecology and Biodiversity, School of Life Sciences, Shandong University, Qingdao, China; ^2^Shandong Provincial Engineering and Technology Research Center for Vegetation Ecology, Shandong University, Qingdao, China; ^3^Qingdao Forest Ecology Research Station of National Forestry and Grassland Administration, Shandong University, Qingdao, China; ^4^Beijing Museum of Natural History, Beijing, China

**Keywords:** leaf damage, leaf morphology, recovery stage, stress tolerance, shade

## Abstract

Seedlings in regenerating layer are frequently attacked by herbivorous insects, while the combined effects of defoliation and shading are not fully understood. In the present study, two Leguminosae species (*Robinia pseudoacacia* and *Amorpha fruticosa*) were selected to study their responses to combined light and defoliation treatments. In a greenhouse experiment, light treatments (L+, 88% vs L−, 8% full sunlight) and defoliation treatments (CK, without defoliation vs DE, defoliation 50% of the upper crown) were applied at the same time. The seedlings’ physiological and growth traits were determined at 1, 10, 30, and 70 days after the combined treatment. Our results showed that the effects of defoliation on growth and carbon allocation under high light treatments in both species were mainly concentrated in the early stage (days 1–10). *R. pseudoacacia* can achieve growth recovery within 10 days after defoliation, while *A. fruticosa* needs 30 days. Seedlings increased SLA and total chlorophyll concentration to improve light capture efficiency under low light treatments in both species, at the expense of reduced leaf thickness and leaf lignin concentration. The negative effects of defoliation treatment on plant growth and non-structural carbohydrates (NSCs) concentration in low light treatment were significantly higher than that in high light treatment after recovery for 70 days in *R. pseudoacacia*, suggesting sufficient production of carbohydrate would be crucial for seedling growth after defoliation. Plant growth was more sensitive to defoliation and low light stress than photosynthesis, resulting in NSCs accumulating during the early period of treatment. These results illustrated that although seedlings could adjust their resource allocation strategy and carbon dynamics in response to combined defoliation and light treatments, individuals grown in low light conditions will be more suppressed by defoliation. Our results indicate that we should pay more attention to understory seedlings’ regeneration under the pressure of herbivorous insects.

## Introduction

The regeneration of woody plant seedlings is an important component of maintaining the vegetation diversity of forest ecosystem (Fukushima et al., 2008). By affecting the long-term succession pattern of forests, it plays a central role in the process of forest dynamic changes (Nyland et al., 2006). In the process of vegetation regeneration, woody plant seedlings often suffer from leaf damage due to herbivores, especially in the context of changing climate conditions (Cannon, 2004; Ballina-Gómez et al., 2010). Among the numerous ecological factors, light conditions and defoliation have an important impact on the survival and regeneration of seedlings (Ballina-Gómez et al., 2010; Chen et al., 2011; Kuehne et al., 2014). Studying the seedlings’ physiological and morphological responses to combined defoliation and light treatments will be helpful to understand the forest dynamics in the regeneration layer and give clues for plant management in restoration activities.

Light, one of the crucial factors for plant photosynthesis and survival, plays a key role in plant growth and development (Chen et al., 2011; Kuehne et al., 2014). As such, the unavailability of light resource often impedes forest regeneration (Baraloto and Forget, 2007). In forest ecosystems, plants usually distribute in various light environments, among which gaps and understory are the most important living environments for regenerating layer seedlings (Wang and Zhou, 2000). Gaps create heterogeneity in understory conditions, which is important for plant regeneration by modifying microclimate and resource availability (Kern et al., 2013). The adaptation of plants to the heterogeneity of the light environment is mainly achieved through different phenotypic plasticity, thereby optimizing the competition and utilization of light resources. Therefore, revealing the physiological and ecological adaptability of the plant seedling stage to light intensity has important practical significance.

Many woody species are frequently attacked by herbivorous insects whatever light environments. Herbivorous insects resulting in complete leaves removal can severely reduce forest productivity, thereby decreasing growth and even causing significant tree mortality (Karolewski et al., 2010). A series of compensatory mechanisms, e.g., increasing leaf photosynthesis, changing leaf morphology and biomass allocation patterns will enhance plant ability to assimilate carbohydrate and maintain growth after defoliation (Quentin et al., 2012; Quijano-Medina et al., 2019; Sanczuk et al., 2020). In addition, previous studies showed that the capacity of plants to recover from defoliation depends to a large extent on resource availability, particularly of light (Żmuda et al., 2008; Piotr et al., 2010). Defoliation and light treatments will affect the translocation effectiveness of carbohydrate to roots, which may affect regeneration of species (Gleason and Ares, 2004). Other study had shown that defoliation caused a decrease in root vitality in *Prunus serotina*, *Cornus sanguinea*, and *Corylus avellana* in low light condition but not in high light condition (Piotr et al., 2010). The carbohydrate reserve dynamics under defoliation and light treatments reveal a trade-off of allocation between growth and storage. Under natural light conditions, most researches show that defoliation leads to an allocation shift, which reduces the priority of growth relative to storage (Smith and Stitt, 2007; Wiley et al., 2013). However, there are still few studies concerning the effect of defoliation and shading on plant carbon allocation patterns.

Leaves are important for gas and heat exchange and carbon gain (Liu et al., 2017). Leaf traits such as specific leaf area (SLA), leaf thickness, chlorophyll concentration, etc., collectively reflect the plants survival and adaptation strategies under shading and defoliation conditions (Houter and Pons, 2012; Paul et al., 2012). Numerous studies indicate that plants grown under low light environment have thinner leaves and lower leaf mass per area (LMA), which goes on the expense of photosynthetic capacity per unit leaf area (Ellsworth and Peter, 1992; Poorter and Bongers, 2006). In addition, other studies have shown that leaves with lower SLA have increased photosynthetic rates, as well as increased physical barriers to herbivores (Salgado-Luarte and Gianoli, 2010; Quentin et al., 2012). Structural traits relevant for defeating biotic constraints include the share of cell walls on leaf biomass and the cell wall composition, with lignin as dominating components, providing mechanical strength against biotic injuries (Hertzberg et al., 2001). Low lignin concentration and leaf thickness reduce the cost of leaf construction, and more resources are invested in photosynthetic organs (Wright et al., 2004).

Understanding carbon allocation patterns within plant holds importance on the scale of individuals. Carbohydrates are usually divided into two types: structural carbohydrates (SCs) and non-structural carbohydrates (NSCs). SCs include lignin, cellulose, semi-fiber, etc., which are involved in plant structure building processes (Niinemets, 1999). NSCs are mainly composed of starch and soluble sugar, which are important energy supply materials in the process of plant growth and metabolism (Dietze et al., 2014). Shading of woody species has a significantly negative impact on the leaf concentration of non-structural carbohydrates (Koricheva et al., 1998; Henriksson et al., 2003). NSCs storage may help plants survive long periods of minimal C gain that may occur sporadically and unpredictably, such as during low light environment or disturbances from herbivores. NSCs would not only reflect the relationship between carbon supply and demand in plants, but also determine the growth of plants (Sheel and Matthew, 2009).

In this study, we explored the effects of artificial defoliation in different light availability on plant growth, leaf traits and carbon allocation of *Robinia pseudoacacia* and *Amorpha fruticosa*. *R. pseudoacacia* is the dominant species in the arbor layer in warm temperate regions of Northern China (Wang and Zhou, 2000). Although *R. pseudoacacia* is listed as an invasive species, it is widely used in the afforestation and vegetation restoration in warm temperate zones for several years (Cierjacks et al., 2013). *R. pseudoacacia* has been planted since the 1980s in China for the purpose of vegetation restoration with approximately 8000 ha planted (Zhang and Xing, 2009). *A. fruticosa* has been planted in the early 20th century in China and is now widely planted throughout the country. *R. pseudoacacia* and *A. fruticosa*, belong to the Leguminosae family and are fast-growing pioneer species (Cierjacks et al., 2013; Guo et al., 2018). They have great nitrogen fixation capacity and resistance to shade, drought and salt stress. Thus, they have prominent ecological functions in soil and water conservation, and maintenance of ecological balance (Dehaan et al., 2006). *R. pseudoacacia* would take active strategies to obtain light resources under light limiting treatments (Xu et al., 2009; Luo et al., 2016), while there are few studies on the responses of *A. fruticosa* to light limitation. Seedling regeneration is an important part of natural succession and vegetation restoration. Both species are frequently attacked by herbivorous insects under various light conditions in the process of vegetation regeneration. In recent years, the numbers and incidence of insect pests associated with *R. pseudoacacia* and *A. fruticosa* have greatly increased (Sharma et al., 2008; Zhang and Xing, 2009; Kolyada and Kolyada, 2019). Leaf-chewing insects such as: *Napocheima robiniae*, *Apogonia cribricollis*, and *Obolodiplosis robiniae* are among the most common pest of two species (Verma et al., 2005; Zhu, 2007; Yao et al., 2015). Previous studies have shown that herbivorous insects feed on new shoots and mostly juvenile leaves (Quentin et al., 2011; Eyles et al., 2013). In this study, we used artificial defoliation to simulate leaf mechanical damage caused by herbivorous insects. We hypothesized that: (1) Seedling growth after defoliation will be significantly suppressed under low light conditions, as the production of carbohydrate will be not enough under shade; (2) Leaf traits and NSC concentration will have sequential responses to combined light and defoliation treatment.

## Materials and Methods

### Plant Material and Experimental Design

The experiment lasted about 5 months from April to September 2018 at the Fanggan Research Station of Shandong University, Shandong Province, China (36°26′N, 117°27′E). The seeds of *R. pseudoacacia* and *A. fruticosa* collected from Shandong Province in the early winter of 2017 were obtained from Qiluyuanyi Seed Company (Linyi, China). The area has a warm temperate monsoon climate, with an annual precipitation of 700 ± 100 mm and an average temperature of 13 ± 1°C. During the experimental period, the microclimate in the greenhouse was monitored with HOBO data loggers (U12-012, Onset, Bourne, MA, United States). Mean air temperature was 29.6°C (18.7–36.7°C) during daytime and 20.8°C (10.2–27.5°C) during nighttime, and mean relative humidity was 59.3% (28.2–97.8%) during daytime and 93.6% (56.3–100%) during nighttime. Seedlings were maintained at 60–70% of field water capacity throughout the experiment utilizing daily irrigation. The seeds were germinated in deionized water on plates and transferred into plastic pots (26 cm in depth and 24 cm in diameter) containing 7 kg of growth substrate (one seedling per pot). The plant growth substrate was a mixture of air-dried sandy loam and humus soils in proportions of 2:3 by volume.

For each species, 3-month-old seedlings of similar size were selected and randomly assigned to the following treatments. For each species, a factorial experiment of two factors (light and defoliation) was designed. The seedlings were randomly assigned to two light conditions: (1) high light treatment (L+), grown in the greenhouse covered by plastic films (88% of natural sunlight); (2) low light treatment (L−), conducted in greenhouse covered by woven black nylon nets (8% of natural sunlight). Under each light treatment, plants were divided into two groups. One group was submitted to top-down 50% defoliation, the upper half leaves were defoliated (DE); and the other was served as a control group without leaf removal (CK). The treatments were conducted from July 12 to September 20, lasting 70 days. There were 16 replicates in each treatment for each species. Four individuals of each species and treatment were randomly selected to measure in each sampling. During the experiment, seedlings were harvested at days 1 (July 13), 10 (July 22), 30 (August 11), and 70 (September 20).

### Growth Measurements

Seedling height and basal diameter (BD, at approximately 1 cm above the ground) were recorded at each harvest. Four seedlings were harvested from each treatment at around noon (12:00–3:00 pm) and separated into roots (include root nodules), stems, and leaves. Then, the samples were oven-dried (30 min at 105°C, followed by 72 h at 75°C) and weighed. Total biomass (TB), leaf mass ratio (LMR), stem mass ratio (SMR), root mass ratio (RMR), and root-shoot ratio (R/S) were calculated as follows:


TB=RB+SB+LB

$$\mathrm{LMR}=\frac{\text{LB}}{\text{TB}}$$

$$\mathrm{SMR}=\frac{\text{SB}}{\text{TB}}$$

$$\mathrm{RMR}=\frac{\text{RB}}{\text{TB}}$$

$$R/S=\frac{\text{RB}}{\text{TB}}$$

where RB is root biomass, SB is stem biomass, and LB is leaf biomass.

### Non-structural Carbohydrate Analysis

Non-structural carbohydrates (NSCs) of four replicates per treatment were measured after each harvest. After biomass determination, dried samples were grounded with a ball mill (JXFSTPRP-24, Jingxin, Shanghai, China) to analyze NSCs (defined as the sum of starch and soluble sugars) concentration in the leaves, stems, and roots (Cao et al., 2018). Soluble sugars (SS) were extracted twice with 80% ethanol, and starch (ST) content was measured after subjecting the solid residue of each sample to a washing step and hydrolysis. The absorbance of the extracts was measured at 620 nm (UV-9000S, Metash, Shanghai, China) after an anthracenone-sulfuric acid reaction. The concentrations of soluble sugars and starch (measured as glucose equivalents) were calculated on dry mass basis (mg g^–1^).

### Leaf Trait Measurements

Gas-exchange characteristics of seedlings were measured before each harvest. The maximum photosynthetic rate (*A*), transpiration rate (*E*), intercellular carbon dioxide concentration (*C*_*i*_), and stomatal conductance (*G*_*s*_) of fully expanded leaves from four replicates (one leaf per seedling) for each measurement were measured using a portable gas exchange measurement system (Li-6800, Li-Cor, Lincoln, NE, United States). These measurements were conducted between 9:00 and 12:00 h on sunny days. During the measurements, photosynthetically active radiation, temperature, relative humidity, and CO_2_ concentration inside the leaf chamber were controlled at 1000 μmol m^–2^ s^–1^, 28°C, 50%, and 400 ppm, respectively. The 1000 μmol m^–2^ s^–1^ PAR was high enough to obtain the maximum photosynthetic rate according to a pre-experiment. Since all the upper half leaves were defoliated, we measured the gas exchange parameters of mature leaves in the middle position of the individuals in all treatments at days 1 and 10. Due to the reflushing leaves were fully expanded at day 30 after defoliation, we measured the gas exchange parameters on newly mature leaves in all treatments at days 30 and 70. Instantaneous water use efficiency (iWUE) was calculated as follows:


$$\mathrm{iWUE}=\frac{A}{E}$$

Specific leaf area (SLA) of four replicates per treatment was also measured after each harvest. Fully expanded and healthy composite leaves (fifty leaflets in total per seedling) from four seedlings in each treatment were scanned, and images were analyzed with the WinFOLIA Pro 2009a software (Regent Instruments, Inc., Quebec, QC, Canada) to determine leaf area. After scanning, these leaves were oven-dried for 72 h at 75°C and weighed. SLA was calculated as leaf area/leaf dry mass. The collection of leaf samples was the same as the gas exchange measurement.

Some other leaf morphological and physiological traits were also measured after 70 days of treatment. Four healthy and fully expanded leaves from the upper reflushing plant part (one leaf per seedling) were selected to determine leaf chlorophyll concentration in each treatment in both species. After extraction with 95% ethanol (v/v), chlorophyll concentration was determined using the spectrophotometric method (Lichtenthaler and Wellburn, 1983). The absorbance (A) was measured at 645 and 649 nm (UV-9000S, Metash, Shanghai, China), Chlorophyll *a* concentration (Chl *a*), Chlorophyll *b* concentration (Chl *b*), the ratio of chlorophyll *a*/*b* (Chl *a*/*b*), total Chlorophyll concentration (Chl_*total*_) were calculated as follows:


$$\text{Chl}\,a=13.95\times A_{665}-6.88\times A_{649}$$

$$\text{Chl}\,b=24.94\times A_{649}-7.32\times A_{665}$$

$$\text{Chl}\,a/b=\frac{\text{Chl}\,a}{\text{Chl}\,b}$$

$${\text{Chl}}_{\text{total}}=\frac{\left(\text{Chl}\,a+\text{Chl}\,b\right)\times \text{V}}{\text{FW}}$$

where V is ethanol volume, and FW is fresh weight.

Leaf thickness was measured with an electronic digital micrometer for four replicates per treatment and was averaged from 20 fresh leaves per seedling. Leaf lignin concentration was estimated by a spectrophotometric procedure using acetyl bromide, and absorbance (A) was measured at 280 nm (UV-9000S, Metash, Shanghai, China). The calculation of the lignin concentration was according to the standard curve (Iiyama and Wallis, 1990).

### Statistical Analysis

Three-way analysis of variance (ANOVA) was applied to detect the effects of light treatment, defoliation treatment and sampling time for plant traits in each species. One-way ANOVA was used to detect difference among treatments for each species, and Duncan’s multiple comparison tests at α = 0.05 were followed when significant differences were observed. Before ANOVA, the data was checked for normality (Shapiro–Wilk test) and homogeneity of variance (Levene test). ANOVA and Spearman correlation was performed using IBM SPSS Statistics 23.0 (IBM Corp., Armonk, NY, United States), and the figures were illustrated using OriginPro 2016 (Originlab Co., Northampton, MA, United States). To evaluate the relationship between plant traits and experimental treatments, redundancy analysis (RDA) was carried out using the *vegan* package in R Statistical Software v.4.0.3 (Oksanen et al., 2019).

## Results

### Combined Treatment Effects on Plant Growth

**FIGURE 1:**
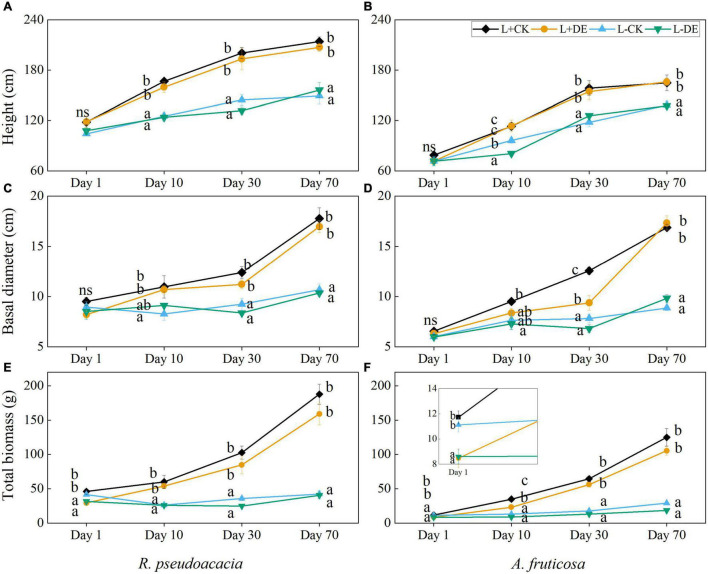
Seedling height, basal diameter, and total biomass of *R. pseudoacacia* **(A,C,E)** and *A. fruticosa* **(B,D,F)** under different light and defoliation treatments. The values are shown as mean ± SE (*n* = 4). Different letters indicate significant differences among different treatments in each sampling according to Duncan’s test (*p* < 0.05). L + CK, high light condition with no defoliation; L + DE, high light condition treatment with defoliation; L−CK, low light condition with no defoliation; L−DE, low light condition with defoliation.

Seedling height, basal diameter and total biomass were affected by time and light treatments, but the interaction of light and defoliation had no significant effect on the growth indexes in both species (Supplementary Tables 1, 2). Owing to the defoliation of upper leaves, the total biomass of the defoliation treatments was significantly lower than control treatments at day 1 in both species (Figures 1E,F). Starting from day 10, the plant height and basal diameter in L+ treatments were higher than L− treatments in both species (Figures 1A,B), and total biomass in defoliation treatments could catch up the seedlings in control in *R. pseudoacacia* (Figure 1E). After 30 days, the total biomass in defoliation treatments could recover to the control level in *A. fruticosa* (Figure 1F). After 70 days, the seedling height, basal diameter and total biomass in L + treatments were all significantly higher than L− treatments in both species (Figure 1).

**FIGURE 2:**
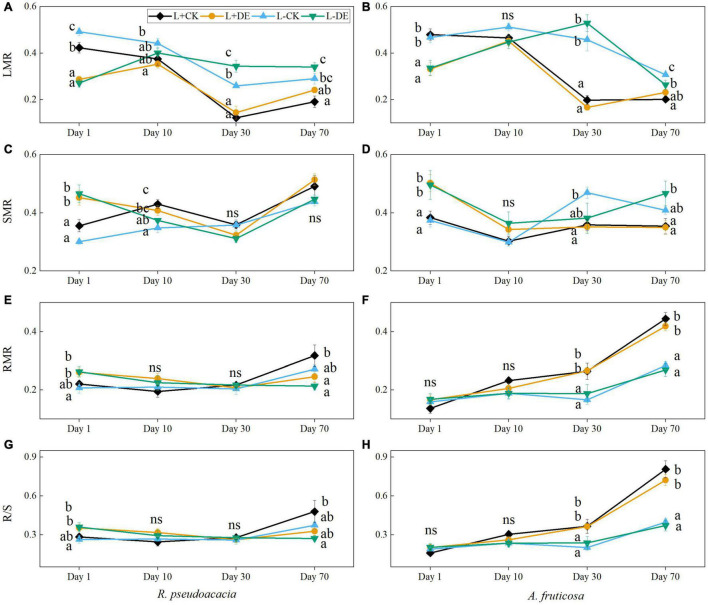
Seedling biomass partitioning parameters of *R. pseudoacacia* **(A,C,E,G)** and *A. fruticosa* **(B,D,F,H)** under different light and defoliation treatments. The values are shown as mean ± SE (*n* = 4). Different letters indicate significant differences among different treatments in each sampling according to Duncan’s test (*p* < 0.05). L + CK, high light condition with no defoliation; L + DE, high light condition treatment with defoliation; L−CK, low light condition with no defoliation; L−DE, low light condition with defoliation.

Defoliation treatment significantly affected the LMR and SMR in both species, light treatment significantly affected the biomass allocation index in *A. fruticosa* (Supplementary Tables 1, 2). The LMR in the L−DE treatment was higher than L−CK treatment at day 30 in *R. pseudoacacia* but not in *A. fruticosa* (Figures 2A,B). Starting from day 30 day, the RMR and R/S in the L+ treatments were higher than L− treatments in *A. fruticosa* but not in *R. pseudoacacia* (Figures 2E–H).

### Combined Treatment Effects on Leaf Traits

**FIGURE 3:**
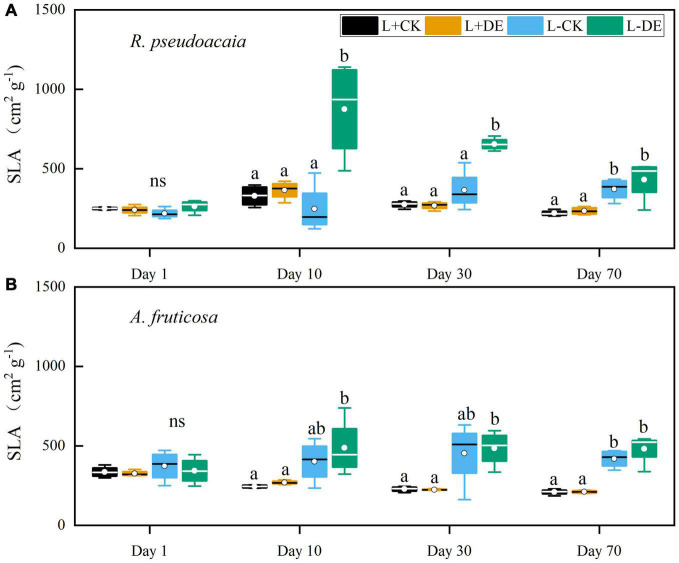
Seedling specific leaf area (SLA) of *R. pseudoacacia* **(A)** and *A. fruticosa* **(B)** under different light and defoliation treatments. The values are shown as mean ± SE (*n* = 4). Different letters indicate significant differences among different treatments at each sampling according to Duncan’s test (*p* < 0.05). L + CK, high light condition with no defoliation; L + DE, high light condition treatment with defoliation; L−CK, low light condition with no defoliation; L−DE, low light condition with defoliation.

Light treatment significantly affected the SLA in both species (Supplementary Tables 1, 2). Defoliation treatment and the interaction of light and defoliation treatment significantly affected the SLA in *R. pseudoacacia* but not in *A. fruticosa* (Supplementary Tables 1, 2). Compared with the L−CK treatment, the SLA of the L−DE treatment increased significantly from day 10–30 in *R. pseudoacacia* (Figure 3A). Compared with the L + DE treatment, the SLA in the L−DE treatment increased significantly during day 10–70 in both species (Figures 3A,B).

**FIGURE 4:**
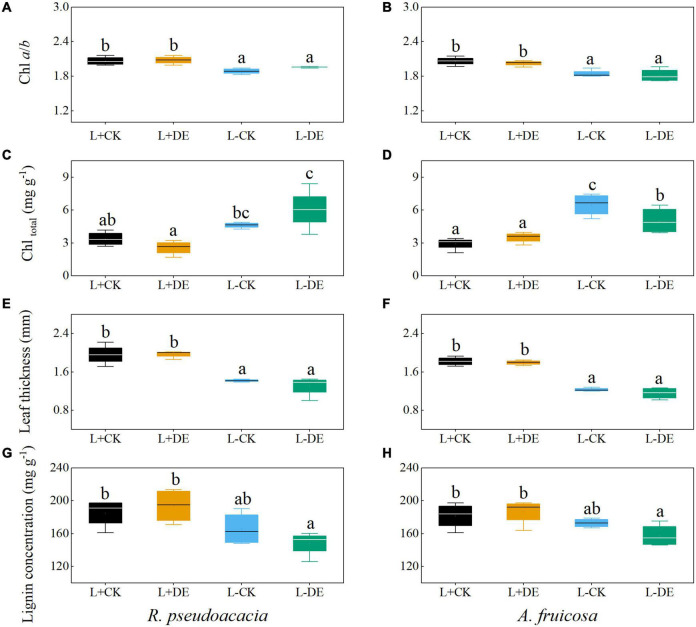
Seedling leaf traits of *R. pseudoacacia* **(A,C,E,G)** and *A. fruticosa* **(B,D,F,H)** under different light and defoliation treatments for 70 days. The values are shown as mean ± SE (*n* = 4). Different letters indicate significant differences among different treatments at each sampling according to Duncan’s test (*p* < 0.05). L + CK, high light condition with no defoliation; L + DE, high light condition treatment with defoliation; L−CK, low light condition with no defoliation; L−DE, low light condition with defoliation.

After 70 days, the Chl *a/b* and leaf thickness in L− treatments were lower than L+ treatments, while the total chlorophyll concentration in L− treatments was higher than L+ treatments in both species (Figures 4A–F). The leaf lignin concentration of L−DE treatment was significantly lower than L + DE treatment in both species (Figures 4G,H).

**FIGURE 5:**
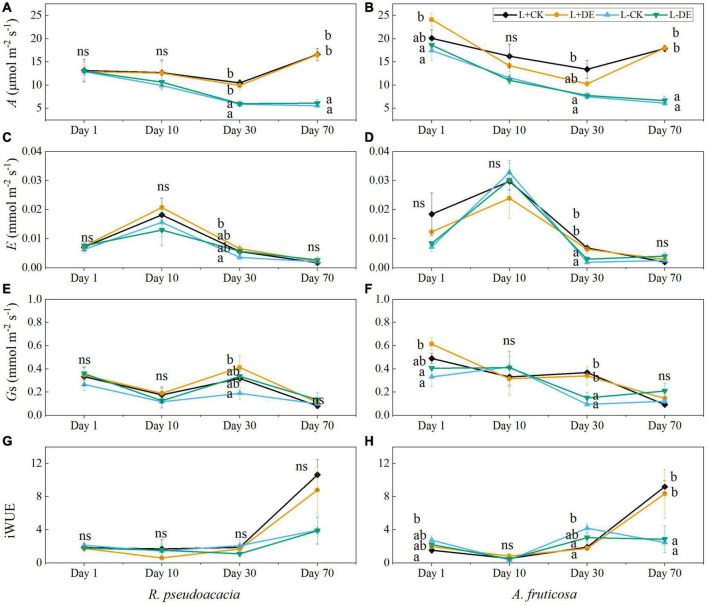
Seedling gas exchange parameters of *R. pseudoacacia* **(A,C,E,G)** and *A. fruticosa* **(B,D,F,H)** under different light and defoliation treatments. The values are shown as mean ± SE (*n* = 4). Different letters indicate significant differences among different treatments in each sampling according to Duncan’s test (*p* < 0.05). L + CK, high light condition with no defoliation; L + DE, high light condition treatment with defoliation; L−CK, low light condition with no defoliation; L−DE, low light condition with defoliation.

Light and time treatments significantly affected the gas exchanges in both species (Supplementary Tables 1, 2). The maximum photosynthetic rate of the L− treatments was lower than L+ treatments starting from day 30 in both species (Figures 5A,B). After 70 days, there was no significant difference in transpiration rate and stomatal conductance among treatments in both species (Figures 5C–F). The iWUE of L− treatments was significantly lower than that of L+ treatments after 70 days in *A. fruticosa* but not in *R. pseudoacacia* (Figures 5G,H).

### Combined Treatment Effects on Carbon Allocation

Light treatment significantly affected the concentration of seedling NSC, stem SS, root SS, leaf ST, and stem ST in both species (Supplementary Tables 1, 2). Defoliation treatment significantly affected the root ST concentration in *R. pseudoacacia* but not in *A. fruticosa* (Supplementary Tables 1, 2). The interaction of light and defoliation treatment significantly affected the root SS and root ST concentration in *A. fruticosa* but not in *R. pseudoacacia* (Supplementary Tables 1, 2).

**FIGURE 6:**
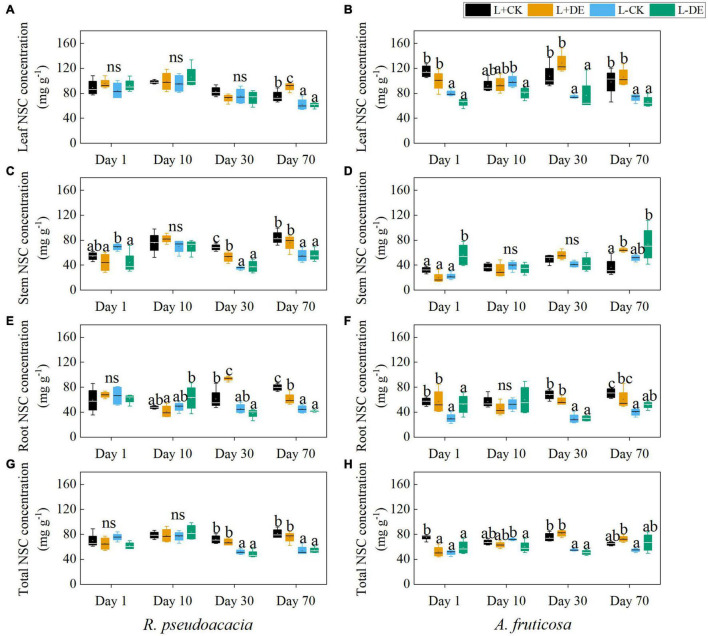
Seedling organs and total non-structural carbohydrate (NSC) concentration of *R. pseudoacacia* **(A,C,E,G)** and *A. fruticosa* **(B,D,F,H)** under different light and defoliation treatments. The values are shown as mean ± SE (*n* = 4). Different letters indicate significant differences among different treatments at each sampling according to Duncan’s test (*p* < 0.05). L + CK, high light condition with no defoliation; L + DE, high light condition treatment with defoliation; L−CK, low light condition with no defoliation; L−DE, low light condition with defoliation.

**FIGURE 7:**
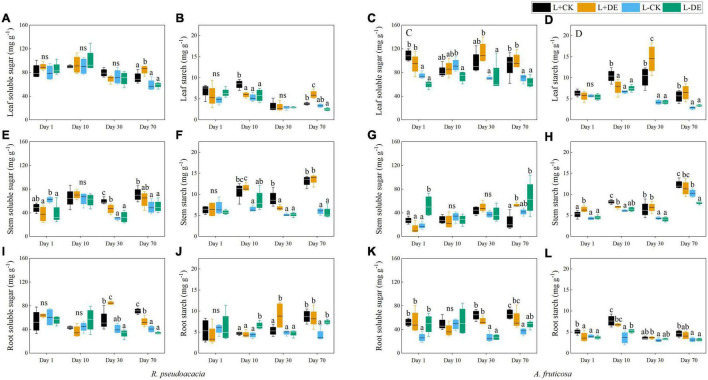
Seedling soluble sugar and starch concentration of *R. pseudoacacia* **(A,B,E,F,I,J)** and *A. fruticosa* **(C,D,G,H,K,L)** under different defoliation and light treatments. Different letters indicate significant differences among different treatments at each sampling according to Duncan’s test (*p* < 0.05). L + CK, high light condition with no defoliation; L + DE, high light condition treatment with defoliation; L−CK, low light condition with no defoliation; L−DE, low light condition with defoliation.

For *R. pseudoacacia*, shading treatment significantly decreased the total NSC concentration in the later stage (days 30–70), but not in the early stage (days 1–10) (Figure 6G). Before the reflushing leaves matured (days 1–10), the leaf ST concentration in defoliation treatment decreased, and the leaf SS concentration remained unchanged (Figures 7A,B). After the new leaves matured (days 30), the leaf NSC concentration in defoliation treatment remained unchanged, the stem NSC concentration was significantly reduced, and the root NSC concentration was significantly increased (Figures 6A,C,E). After 70 days, the stem SS and ST concentration, root SS and ST concentration in L−CK treatment were significantly lower than L + CK treatment (Figures 7E,F,I,G).

For *A. fruticosa*, defoliation and light treatment significantly decreased seedling total NSC concentration at day 1 (Figure 6H). After 10 days, there was no significant difference in leaf, stem and root NSC concentration between L + CK treatment and L−CK treatment (Figures 6B,D,F). After 30 days, the leaf and stem ST concentration in L−CK treatment were significantly lower than L + CK treatment, the leaf and stem SS concentration remained unchanged (Figures 7C,D,G,H). After 70 days, the root SS and ST concentration in L−CK treatment were significantly lower than L + CK treatment (Figures 7K,L).

### Relationships and Trade-Offs Among Plant Traits

**FIGURE 8:**
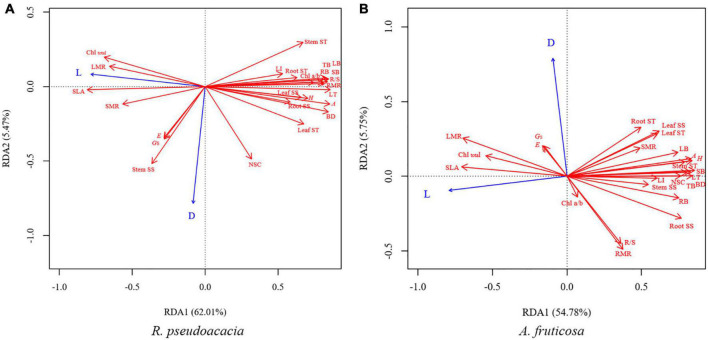
Redundancy analysis (RDA) of the effects of light treatments (L) and defoliation (D) on plant traits of *R. pseudoacacia* **(A)** and *A. fruticosa* **(B)** after treatment for 70 days. *H*, height; BD, basal diameter; TB, total biomass; LB, leaf biomass; SB, stem biomass; RB, root biomass; LMR, leaf mass ratio; SMR, stem mass ratio; RMR, root mass ratio; R/S, root-shoot ratio; SLA, specific leaf area; *A*, the maximum photosynthetic rate; *E*, transpiration rate; *G*_*s*_, stomatal conductance; Chl_*total*_, total chlorophyll concentration; Chl *a/b*, Chlorophyll *a/b*; LT, leaf thickness; LI, leaf lignin concentration; Leaf SS, leaf soluble sugar concentration; Stem SS, stem soluble sugar concentration; Root SS, root soluble sugar concentration; Leaf ST, leaf starch concentration; Stem ST, stem starch concentration; Root ST, root starch concentration; NSC, non-structural carbohydrates concentration.

Redundancy analysis was performed for both species after 70 days. The first two axes explained 67.48% of the variation in *R. pseudoacacia* (Figure 8A), and 60.53% of the variation in *A. fruticosa* (Figure 8B). SLA was positively correlated with total chlorophyll concentration, while negatively correlated with the leaf thickness and leaf lignin concentration. Low light treatment was positively correlated with SLA and total chlorophyll concentration, but negatively correlated with maximum photosynthetic rate, leaf thickness, leaf lignin concentration, and growth parameters in both species. Defoliation treatment was negatively correlated with RMR and R/S in *R. pseudoacacia*, while positively correlated with stomatal conductance (Figure 8).

## Discussion

### Seedlings Were More Significantly Suppressed Under Low Light Conditions After Defoliation

After 70 days of recovery, compensatory growth in response to defoliation was observed in both species, with a greater compensatory total biomass growth in high light versus low light availability treatments. Our results corroborate previous research in other species, which showed that moderate (50–66%) defoliation did not affect growth in *Nothofagus solandri* var. *cliffortioides* seedlings (Mikola et al., 2000). However, low light treatments after 70 days significantly altered growth, and leaf morphological and physiological parameters of both species. Plant height, basal diameter, total biomass, maximum photosynthetic rate, Chlorophyll *a/b*, and leaf thickness all decreased in low light treatments, while SLA and total leaf chlorophyll concentration increased (Supplementary Tables 1, 2 and Figures 1, 3–5). These results showed that plants can undergo plastic changes in morphology and physiological characteristics to adapt to changing light conditions, which is also an important way for plants to adapt to environmental heterogeneity (Õunapuu-Pikas and Sellin, 2020).

In our study, SLA was positively correlated with total chlorophyll concentration in both species (Figure 8). SLA is a major trait in the worldwide leaf economics spectrum, which reflects nutrient and dry mass investment in leaves (Wright et al., 2004). A higher SLA and total chlorophyll concentration grown under low-light conditions could help plants increase the efficiency of light capture and maximize carbon gain by reducing the diffusion resistance of CO_2_, leaf construction and maintenance costs and the self-shading of the chloroplast (Rijkers et al., 2000; Xu et al., 2009; Gommers et al., 2013; Takahashi and Obata, 2014; Freschet et al., 2015). Other studies suggest that low chlorophyll *a/b* is considered to be a characteristic of plant’s shade tolerance, which also could improve the light-capture efficiency in low light environment (Hansen et al., 2002). The plastic response of SLA to shading may result in thinner, and relatively larger, leaves (Liu et al., 2016). In our study, results in low light treatment were consistent with leaf cost-benefit strategies (Zhu et al., 2016). Seedlings reduced leaf structural investment by reducing leaf thickness and leaf lignin concentration, thereby increasing SLA and total chlorophyll concentration to increase the investment in light efficiency capture (Figures 3, 4), which suggested that plants could achieve high carbon gain at a low leaf carbon cost under low light conditions. The RDA analysis also proved that there was a trade-off between leaf structural investment and light capture efficiency during low light treatments (Figure 8). The increase in SLA under low light conditions was concurrent with declines in leaf thickness and leaf lignin concentration, which was also an adaptation strategy in low light conditions to achieve high carbon gain.

This experiment demonstrated that plant growth and NSC concentration were reduced in L−DE treatment compared with L + DE treatment after 70 days in *R. pseudoacacia* (Figures 1, 6). The result supports our first hypothesis that seedling growth after defoliation would be significantly suppressed under low light conditions, as the production of carbohydrate is not enough. However, in the early period, plant growth was also reduced in L−CK treatment compared with L + CK treatment, although the NSC concentrations were maintained at control levels after treatments for 10 days in *R. pseudoacacia* (Figures 1, 6). The results showed that growth suppression might not be caused by insufficient supply of NSCs. The previous study showed that plant growth was more sensitive to stress than photosynthesis, resulting in NSC accumulating (Weber et al., 2019). Thus, the decrease of NSC concentration as an indicator of C limitation is not accurate.

### Sequential Responses of Plant Traits to Defoliation and Light Treatment

In the early experiment (days 1–10), we found that stems soluble sugar concentration decreased significantly after defoliation, while the leaves soluble sugar concentration remained stable in *R. pseudoacacia* at day 1 (Figure 7). After removing the upper leaves, the production of new leaves requires a lot of carbohydrates, thereby remobilizing carbon stored in the stems. After 10 days, the leaves starch tended to be more depleted in defoliation treatment compared with controls in both species (Figure 7), indicating that leaf flush relies on stored NSC reserves (Merryn et al., 2018). Meanwhile, under low light treatment, defoliation significantly decreased LMR and increased SLA, but did not significantly affect RMR in *R. pseudoacacia* at day 10 (Figures 2, 3), suggesting that seedlings adapt to defoliation and light stress in the early stage by changing leaf morphology and physiological characteristics rather than root growth. From the gas exchange characteristics, *A. fruticosa* was a species with high rate of photosynthesis and transpiration. The transpiration rate and stomatal conductance of L− treatments were higher than L+ treatments, but there was no significant difference in the maximum photosynthetic rate between L+ treatments and L− treatments at day 10 (Figure 6). Previous study had shown that shade-tolerant species often retained open stomata in low light conditions (Hetherington and Woodward, 2003). The carbon limitation caused by L + DE or L−CK did not change the NSC concentration in *A. fruticosa*, but the L−DE treatment reduced the NSC concentration. This indicated NSC levels would not decrease as growth declined under moderate carbon limitation, but as limitation became severe, NSC levels would decline (Erin et al., 2013), which also showed that defoliation had a greater impact on seedlings under low light conditions.

In the late phase of the experiment (days 30–70), growth and carbon allocation recovered rapidly after defoliation under high light conditions but not under low light conditions in both species. With the prolongation of the shading stress, the plant NSC concentration, height, basal diameter, total biomass, and maximum photosynthetic rate in the L− treatments was lower than that of L+ treatments in both species at day 30. Thus, shade stress can inhibit photosynthesis and cause insufficient carbon supply, resulting in carbon limitation, and limited plant growth (Sala et al., 2012), which was consistent with previous study that plants reduced their photosynthetic capacity in response to low light availability (Rossatto et al., 2010). In addition, the NSC concentration in different organs was consistent with their respective functions (Figure 6). Under moderate defoliation, the reduction in growth showed significant trend of recovery over time. However, under low light treatments, the plant height, basal diameter and total biomass were smaller in both species after 70 days compared to the high light treatment (Figure 1). The results showed that insufficient carbon supply would inhibit plant growth and carbon storage (Ida et al., 2012). However, there was no significant difference in the total NSC concentration in L−CK treatment compared with the control after 70 days in *A. fruticosa*, but the growth parameters of L−CK treatment was significantly lower than control group. As an adaptation to increasing carbon limitation, plants can prioritize maintaining NSC concentration at the expense of growth, which could help plants survive long periods of minimal C gain (Erin et al., 2013; Elise and Jason, 2020).

Previous studies showed that herbivore damage could vary significantly between the contrasting light habitats (open vs. shade) on various species (Miljković et al., 2018). Under high light conditions, defoliation treatment had a significant impact on physiological indicators and carbon allocation during the early recovery period (days 1–10), but in the later recovery period (days 30–70), defoliation had little effect on plant growth and physiological characteristics. Under low light conditions, defoliation treatment first reduced the growth parameters, SLA, and then reduced the plant’s gas exchange parameters and total NSC levels. In the early stage of the experiment, defoliation and light treatment had a significant impact on leaf physiological and morphological traits, and less on the root growth. As the stress continued, the root growth was also severely restricted in the later stage of the experiment. Previous studies have shown that adaptation of plant to variation in light during herbivore activity depends on plant resource allocation (Piotr et al., 2010).

### Different Recovery Strategies of *R. pseudoacacia* and *A. fruticosa* Seedlings

It has been shown that *R. pseudoacacia* seedlings can recover their total biomass in about 10 days after defoliation under high light condition, but *A. fruticosa* seedlings could catch up their total biomass with control within 30 days (Figure 1). These results showed that the *R. pseudoacacia* seedlings recovered faster than *A. fruticosa* seedlings after defoliation. Low light treatments significantly altered the growth and biomass allocation strategy of *A. fruticosa*. LMR in L− treatment was significantly higher than the L+ treatment at day 30, while RMR in L− treatment was significantly lower than the L+ treatment in *A. fruticosa* (Figure 2), which indicated that plant invested more carbohydrate into the growth of aboveground organs than of roots, consistent with the functional equilibrium hypothesis that resources are allocated to organs which are responsible for acquiring the most limiting resource (Poorter et al., 2012).

## Conclusion

Our results indicated that *R. pseudoacacia* seedlings recovered faster than *A. fruticosa* seedlings after defoliation under high light condition. Low light treatment could inhibit plant growth and carbon storage in both species. Growth after defoliation would be significantly suppressed under low light conditions, as the production of carbohydrate was not sufficient after 70 days in *R. pseudoacacia*. Plants can prioritize maintaining NSC concentration at the expense of growth under carbon limitation in *A. fruticosa*, which could help plants survive long periods of minimal C gain. Future researches should increase field experiments to have a more comprehensive understanding of the adaptation strategies of regenerating layer seedlings to complex habitats.

## Data Availability Statement

The raw data supporting the conclusions of this article will be made available by the authors, without undue reservation.

## Author Contributions

NW conducted the experiment, wrote the body of the manuscript, and performed sample preparations, and laboratory and data analyses. PZ set up the experimental design and provided funding. TJ and KS performed the experiments. XL, QL, PW, and HS conducted analyses. ND, HW, and RW contributed in editing the manuscript. All authors made a contribution to this work.

## Conflict of Interest

The authors declare that the research was conducted in the absence of any commercial or financial relationships that could be construed as a potential conflict of interest.

## Publisher’s Note

All claims expressed in this article are solely those of the authors and do not necessarily represent those of their affiliated organizations, or those of the publisher, the editors and the reviewers. Any product that may be evaluated in this article, or claim that may be made by its manufacturer, is not guaranteed or endorsed by the publisher.
