# Supplementary material for: Defoliation Significantly Suppressed Plant Growth Under Low Light Conditions in Two Leguminosae Species
Source: Front Plant Sci. 2022 Jan 7;12:777328. doi: 10.3389/fpls.2021.777328 (PMC8776832; doi:10.3389/fpls.2021.777328)
Supplement: Supplementary file 1 [file Table_1.DOCX]

**Table S1** Three-way ANOVA from harvest times (days 1, 10, 30, and 70), light (low light versus high light), defoliation (defoliation versus non-defoliation), and their interactions on plant traits for *R. pseudoacacia* seedlings.

| Parameters | *R. pseudoacacia* | | | | | |  |
| --- | --- | --- | --- | --- | --- | --- | --- |
|  | T | L | D | T×L | T×D | L×D | T×L×D |
| *H* (cm) | **88.617***** | **168.532***** | 0.849 | **11.390***** | 0.661 | 0.443 | 0.383 |
| BD (mm) | **54.601***** | **99.117***** | 3.155 | **21.491***** | 0.927 | 1.289 | 0.090 |
| TB (g) | **69.440***** | **218.135***** | **9.022**** | **53.144***** | 0.539 | 2.248 | 0.449 |
| LMR | **70.764***** | **78.980***** | **7.163*** | **9.643***** | **30.121***** | 0.274 | 2.295 |
| SMR | **32.592***** | **13.894**** | **7.546**** | 1.899 | **14.326***** | 1.383 | 1.161 |
| RMR | **6.815**** | 1.749 | 0.140 | 1.113 | **7.982***** | 0.105 | 0.475 |
| R/S | **6.287**** | 1.786 | 0.009 | 1.292 | **7.463***** | 0.233 | 0.393 |
| *A* (μmol m^-2^ s^-1^) | **7.820***** | **34.476***** | 0.01 | **9.615***** | 0.028 | 0.166 | 0.006 |
| *E* (mmol m^-2^ s^-1^) | **26.670***** | 1.804 | 0.353 | 1.019 | 0.074 | 0.092 | 0.378 |
| *G*_s_ (mmol m^-2^ s^-1^) | **15.517***** | 2.246 | 3.709 | 0.835 | 0.694 | 0.346 | 0.124 |
| iWUE | **21.629***** | **6.001*** | 1.007 | **6.968**** | 0.072 | 0.189 | 0.299 |
| SLA (cm^2^ g^-1^) | **12.568***** | **36.349***** | **26.135***** | **4.493**** | **7.791***** | **22.896***** | **6.269**** |
| Leaf SS (mg g^-1^) | **16.372***** | 3.467 | 1.394 | **2.970*** | 1.432 | 0.002 | 0.736 |
| Stem SS (mg g^-1^) | **14.310***** | **10.444**** | 4.781 | **5.335**** | 1.967 | 0.031 | 1.539 |
| Root SS (mg g^-1^) | **4.506**** | **20.472***** | 0.096 | **14.536***** | **3.3000*** | 1.101 | **5.583**** |
| Leaf ST (mg g^-1^) | **30.253***** | **15.182***** | 0.010 | 2.064 | 1.457 | 1.698 | **6.132**** |
| Stem ST (mg g^-1^) | **26.670***** | **104.778***** | 0.010 | **20.643***** | 2.569 | 0.353 | 1.483 |
| Root ST (mg g^-1^) | **4.268**** | 2.756 | **4.137*** | **4.898**** | 0.973 | 1.055 | **3.074*** |
| NSC (mg g^-1^) | **14.570***** | **23.253***** | 2.331 | **10.116***** | 1.514 | 0.095 | 0.716 |

Notes: T, harvest time; L, light treatment; D, defoliation treatment; *H*, height; BD, basal diameter; TB, total biomass; LB, leaf biomass; SB, stem biomass; RB, root biomass; LMR, leaf mass ratio, SMR, stem mass ratio, RMR, root mass ratio, R/S, root-shoot ratio; SLA, specific leaf area; *A*, the net photosynthetic rate, *E*, transpiration rate, *G*_s,_ stomatal conductance; Leaf SS, leaf soluble sugar concentration; Stem SS, stem soluble sugar concentration; Root SS, root soluble sugar concentration; Leaf ST, leaf starch concentration; Stem ST, stem starch concentration; Root ST, root starch concentration; NSC, non-structural carbohydrate concentration. *, *p* < 0.05; **, *p* < 0.01; ***, *p* < 0.001. × represents the interaction effect. *n* = 4.

**Table S2** Three-way ANOVA from harvest times (days 1, 10, 30, and 70) light (low light versus high light) and defoliation (defoliation versus non-defoliation), and their interactions on plant traits for *A. fruticosa* seedlings.

| Parameters | *A. fruticosa* | | | | | |  |
| --- | --- | --- | --- | --- | --- | --- | --- |
|  | T | L | D | T×L | T×D | L×D | T×L×D |
| *H* (cm) | **164.124***** | **66.064***** | 0.674 | **5.747**** | 0.574 | 0.002 | 1.206 |
| BD (mm) | **92.721***** | **130.418***** | **6.267*** | **94.379***** | 2.620 | 2.031 | 0.901 |
| TB (g) | **168.346***** | **369.176***** | **16.438***** | **97.103***** | 1.659 | 1.628 | 0.208 |
| LMR | **59.346***** | **67.745***** | **11.677**** | **35.499***** | **8.316***** | 0.002 | 2.685 |
| SMR | **10.066***** | **7.294*** | **6.932*** | 2.483 | **5.676**** | 0.007 | 1.092 |
| RMR | **80.647***** | **48.671***** | 0.007 | **14.871***** | 1.034 | 0.241 | 0.320 |
| R/S | **92.722***** | **62.244***** | 0.208 | **24.276***** | 1.153 | 0.623 | 0.282 |
| *A* (μmol m^-2^ s^-1^) | **39.114***** | **70.044***** | 0.006 | **6.949**** | 1.762 | 0.192 | 0.855 |
| *E* (mmol m^-2^ s^-1^) | **53.826***** | 0.897 | 0.631 | 2.744 | 0.587 | 0.933 | 0.206 |
| *G*_s_ (mmol m^-2^ s^-1^) | **15.386***** | 3.800 | 1.453 | **5.097**** | 0.505 | 0.084 | 0.153 |
| iWUE | **22.154***** | **4.403*** | 0.151 | **14.728***** | 0.148 | 0.051 | 0.331 |
| SLA (cm^2^ g^-1^) | 0.385 | **43.415***** | 1.351 | **4.102*** | 0.443 | 1.095 | 0.318 |
| Leaf SS (mg g^-1^) | 0.698 | **48.163***** | 0.791 | **4.859**** | 2.129 | 3.437 | 0.463 |
| Stem SS (mg g^-1^) | **9.848***** | **4.208*** | **8.967**** | **3.516*** | **4.762**** | 2.425 | **4.573**** |
| Root SS (mg g^-1^) | 1.608 | **23.600***** | 0.237 | **7.546***** | 1.496 | **8.121**** | 0.172 |
| Leaf ST (mg g^-1^) | **25.078***** | **87.558***** | 1.098 | **21.209***** | **3.889*** | 0.069 | **5.054**** |
| Stem ST (mg g^-1^) | **121.141***** | **79.532***** | 1.861 | 2.571 | **4.816**** | 0.695 | 2.286 |
| Root ST (mg g^-1^) | **33.695***** | **46.486***** | 0.545 | **7.923***** | 1.851 | **7.663**** | 2.111 |

Notes: T, harvest time; L, light treatment; D, defoliation treatment; *H*, height; BD, basal diameter; TB, total biomass; LB, leaf biomass; SB, stem biomass; RB, root biomass; LMR, leaf mass ratio, SMR, stem mass ratio, RMR, root mass ratio, R/S, root-shoot ratio; SLA, specific leaf area; *A*, the net photosynthetic rate, *E*, transpiration rate, *G*_s,_ stomatal conductance; Leaf SS, leaf soluble sugar concentration; Stem SS, stem soluble sugar concentration; Root

SS, root soluble sugar concentration; Leaf ST, leaf starch concentration; Stem ST, stem starch concentration; Root ST, root starch concentration; NSC, non-structural carbohydrate concentration. *, *p* < 0.05; **, *p* < 0.01; ***, *p* < 0.001. × represents the interaction effect. *n* = 4.
